# Label Information About Fermentation Processing Affects Consumers’ Sensory and Hedonic Judgements of Specialty Coffee

**DOI:** 10.3390/foods15081287

**Published:** 2026-04-09

**Authors:** Fabiana M. Carvalho, Maísa M. M. de Sousa, Denis Henrique S. Nadaleti

**Affiliations:** 1 Department of Food Science and Nutrition, Faculty of Food Engineering, State University of Campinas (UNICAMP), Rua Monteiro Lobato 80, Cidade Universitária, Campinas 13083-862, SP, Brazil; 2 Southern Minas Regional Unit, Agricultural Research Company of Minas Gerais (Epamig), Lavras 37203-200, MG, Brazil; maisamancini@gmail.com (M.M.M.d.S.); denisfitotecnia@gmail.com (D.H.S.N.)

**Keywords:** specialty coffee, label information, flavour, consumer

## Abstract

Coffee label information impacts consumer choice by communicating key product attributes. This study investigated whether label information on fermentation-related post-harvest processing techniques influence specialty coffee consumers’ expectations and perception of brewed coffee. A total of 180 specialty coffee consumers participated in a within-subject tasting experiment, evaluating the same coffee paired with three labels: no processing information, ‘fermentation’, and ‘carbonic maceration’. Participants first rated their expectations of aroma, flavour, acidity, sweetness, and subsequently, their experience of those attributes on tasting the coffees, as well as rating their liking and purchase intent. Additionally, they also assessed the usual-to-exotic flavour expectation and perceived price of coffees processed with traditional and innovative post-harvest methods. Results showed that the coffee paired with the label ‘fermentation’ was expected to be the most acidic and the least liked, which was confirmed during tasting, whereas the label ‘carbonic maceration’ increased curiosity and perceived novelty towards the coffee without negatively affecting the sensory acceptance. Innovative fermentation-related terms were also perceived as more exotic and expensive compared to traditional methods. These findings demonstrate that descriptive post-harvest terms on coffee labels significantly influence consumer expectations, sensory perception, and perceived value. They also highlight the importance of carefully selecting labelling terms to balance consumer curiosity, sensory expectations, and product acceptance.

## 1. Introduction

Fermentation is one of the oldest methods of food processing. It can be broadly defined as a bioprocess that utilizes microorganisms and their enzymes to achieve desirable quality characteristics in food. Fermentation can be used for food preservation, improving nutritional value, and enhancing sensory attributes [1].

The fermentation itself is part of a larger manufacturing process of a food product. For cocoa and coffee processing, for example, spontaneous fermentative steps are crucial at post-harvest to remove the sticky mucilage (pulp) surrounding the beans. For coffee in particular, the post-harvest processing of the coffee cherry is done using three different types of fermentation techniques, known as dry, wet, or semi-dry [2]. In addition to the pulp removal, fermentation also triggers an array of chemical changes within the beans, which in turn changes the compounds formed during roasting and the coffee sensory profile [3].

### 1.1. The Specialty Coffee Segment

Coffee is one of the most commonly consumed beverages worldwide. Traditionally, coffee has been traded as a commodity and consumed mainly in industrial blends [4]. However, over the past years, the prospect of coffee trading and consumption has changed significantly, with consumers increasingly seeking higher quality coffee and sensory profile differentiation. This shift has led to the emergence of new coffee consumption trends, particularly the growing popularity of specialty coffees [5,6]. The Specialty Coffee Association (SCA) has defined standardized methodologies to assist buyers and producers in evaluating the sensory quality of their products, especially for fair and more attractive trade [7,8].

Recognizing the impact of fermentative processes on the sensory qualities of coffee, producers are striving to develop new post-harvest practices that increase sensory diversification to meet the market demands and add value to their product. Indeed, coffee post-harvest processes have evolved considerably over the years [9]. A key focus has been put on conducting the fermentation steps at the farm in a controlled manner, particularly through the use of starter cultures and the addition of carbon dioxide (CO_2_) [3]. Thus, in addition to the traditional methods described as ‘natural’ and ‘washed’, multiple nomenclatures have emerged to describe innovative fermentation processes, such as ‘carbonic maceration’, ‘anaerobic’, and ‘yeast fermentation’ [10].

The coffee supply chain is complex and involves various stages related to the buying and selling of both green and roasted coffee in business-to-business (B2B) and business-to-consumer (B2C) markets [11]. One key distinction between B2B and B2C contexts is the different factors influencing purchasing decisions. In the B2B context, information about post-harvest processing methods serves as a predictor of specialty coffee prices since it is considered a proxy for quality by green coffee buyers [12,13,14]. Typically, in the specialty coffee market, much of the relevant B2B information about green coffee—such as origin, plant variety, and post-harvest processing—is included on retail (B2C) coffee labels. As suggested by [15], this practice reflects the commitment of coffee roasters to ensure traceability within their supply chains, which contributes to the differentiation of their coffee products. Research has shown that label details regarding origin, roast level, and sensory descriptors are associated with consumer preferences, indicating that they are generally interpreted correctly [16,17,18,19]. However, to the best of our knowledge, no studies have directly examined whether label information related to coffee fermentative processes influences consumers’ sensory and hedonic judgments of coffee, as well as their coffee choices.

### 1.2. Study Rationale

It has been widely reported that product-extrinsic sources of information—which are not physically a part of the product but are somehow related to it—largely influence consumers’ expectations towards the food product itself. Product-extrinsic cues include elements of packaging, marketing, and labelling including any verbal terms used to describe or communicate a food product and can, in turn, generate expectations and alter product perception [20,21].

One increasingly popular theoretical framework to explain how our brains use expectations to make sense of new sensory stimuli is in terms of predictive processing [22,23]. According to this theory, perception is not a simple and passive analysis of sensory input. Instead, it states that our perceptions are formed by integrating stored information from our prior experiences (in the form of expectations or predictions) with the current, actual stimuli. This also applies to food and eating behaviour. Thus, the consumer’s brain is not passively waiting to be activated by sensory data at the moment of consumption; rather, it is proactive in anticipating the upcoming stimulation based on memory-based expectations [24,25]. Moreover, the consumer’s actual perception or experience can either match or mismatch these expectations. A mismatch between predicted and perceived sensory stimuli generates what is called prediction error. The minimization of such errors can follow two different patterns of expectation effects—contrast and assimilation—depending on whether the prediction error was large or small, respectively [26,27]. Contrast is a bias that magnifies the difference between prior expectation and posterior experience whereas assimilation is a bias that diminishes expectation incongruence. Both patterns of prediction error minimization have been shown to impact consumer satisfaction towards various products [28].

This study investigated whether indicating coffee fermentation techniques on bag labels influences consumers’ expectations and actual perception of specialty coffee. There is a large amount of evidence that food process-related terms significantly influence consumers’ behaviour towards food and can also impact their perception of it [29,30,31]. Regarding the term ‘fermentation’ specifically, research shows that fermented foods are strongly associated with sour taste [32,33]; indeed, some of these beverages/foods even carry the attribute as part of their commercial names (e.g., sourdough bread, sauerkraut, sour beer).

Based on this evidence, we developed a few hypotheses. First, participants would expect higher acidity in coffee samples labelled with terms containing ‘fermentation’ compared to those without descriptors or with alternative and vague descriptions such as ‘carbonic maceration’. Second, we hypothesized that heightened expectations of acidity primed by the term ‘fermentation’ would carry over to influence actual perception of the acidity level in the tasted coffee through an assimilation process.

Finally, it has been suggested that consumers can perceive foods produced with more complex and innovative processes as being of higher quality and having unique flavours [34,35]. Thus, we also explored whether novel fermentation-related terms, such as ‘carbonic maceration’, ‘yeast fermentation’ and ‘anaerobic fermentation’, are perceived as more expensive and more exotic than traditional fermentation techniques.

Retail label information on food credence attributes spans categories related to production methods, origin, and certification systems, as well as health, environmental, and social orientation. This information greatly influences consumers’ attitudes toward food (see [36] for a comprehensive review). In the context of coffee, previous research has demonstrated that credence attributes on labels—such as country or region of origin [17,37], environmental claims (e.g., ‘eco-friendly’ [38]), and brand [39]—can influence consumers’ sensory evaluation of coffee. This study breaks new ground by examining how information about post-harvest methods, particularly details about novel fermentative processes, affects consumer sensory perception and purchasing decisions for specialty coffee, a rapidly growing market.

## 2. Materials and Methods

### 2.1. Participants

A total of 180 participants gave their informed consent to take part in the tasting session (Female: 51.7%, Male: 48.3%; Age range 18–62 years, mean = 35.9; SD = 10.7). The eligibility criterion was being an amateur coffee consumer (as opposed to a professional one) who had been purchasing and drinking specialty coffee for at least a year. All participants were recruited through social media profiles of specialty coffee shops in Sao Paulo. The study was approved by the Research Ethics Committee of the Federal University of Lavras, Lavras, Brazil (CAAE 86876125.4.0000.5148).

### 2.2. Stimuli

#### 2.2.1. Coffee

A single-origin Arabica coffee was used in the study. The green coffee beans came from the micro-lot of a production plot (900–950 m of altitude) EPAMIG Experimental Farm located in Três Pontas, South of Minas Gerais, Brazil. The cultivar was MG Paraiso 2, and the post-harvest processing method consisted of anaerobic fermentation for 96 h using *Saccharomyces cerevisiae* [40]. The coffee was roasted in a Stratto^®^ (Carmomaq Exceptional Roasters, Espírito Santo do Pinhal, Brazil), according to the Specialty Coffee Association protocol [41] in order to achieve a light-to-medium roast profile (60–55 Agtron units—Agtron, Inc., Reno, NV, USA).

The coffee sample was graded by six SCA-certified sensory analysts (Qgraders) and was chosen based on its medium scores in intensity scales for aroma, acidity, sweetness, and body, as well as its overall score of 85 points. According to the SCA evaluation [41], grades on a scale of zero to ten are awarded for the attributes of fragrance/aroma, sweetness, acidity, body, flavour, balance, and aftertaste. A 100-point scale is then used to summarize the flavour and esthetic qualities of a brewed coffee. Specialty coffees are those that receive a cupping score of 80 points or higher. The coffee used in this experiment scored 85 points, indicating that it has no primary defects (i.e., dominant bitterness and astringency) and features a balanced sweetness-to-acidity ratio. Regarding overall aroma/flavour characteristics, the coffee was described as having balanced sweetness-to-acidity with prominent fruit notes resembling yellow tropical fruits and medium body.

The coffee beverages served to the participants were brewed as a drip (pour-over) coffee using the Hario V60 Kit (Hario V60, Tokyo, Japan) at a concentration of 77 g·L^−1^ obtained by pouring hot mineral water (94 °C) over the roasted and ground coffee [42]. The coffees were brewed by a professional barista out of the participants’ sight. The mean temperature of coffee served to the participants was 62.5 °C (SD = 0.9).

#### 2.2.2. Packaging Label Information

All three labels had the same information on variety and region of origin, but differed in information on the fermentative post-harvest process. One label featured no post-harvest information at all (‘no information’—control condition. It is important to note that a large body of research has demonstrated that the mere presence of packaging significantly influences our perception of food and beverages [43,44]. Taking this into account, a second control condition featuring only the coffee sample was intentionally excluded from the study. The primary objective of the experiment was to investigate the impact of label information on coffee tasting, rather than to assess the overall influence of packaging (i.e., absence versus presence). The other two labels provided information about the fermentative post-harvest process in two ways: one simply stated ‘fermentation’, while the other used the term ‘carbonic maceration’. These terms were selected to create two different levels of informativeness carried by the descriptor. The term ‘fermentation’ is accurate whilst the term ‘carbonic maceration’ is vague. Thus, the label ‘fermentation’ can be regarded as being more informative than the label ‘carbonic maceration’ when it comes to the putative sensory attributes of the coffee [24,45].

**Figure 1:**
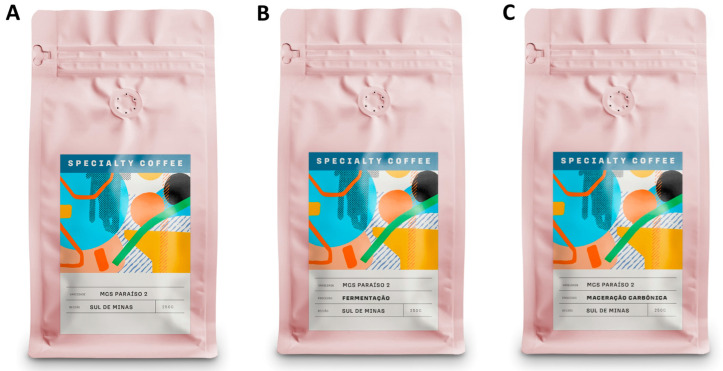
The three different coffee packaging labels used in the experiment. (**A**) No information, (**B**) Fermentation, (**C**) Carbonic maceration. Label information is in Portuguese as the experiment was conducted in Brazil with Brazilian consumers. *Variedade*—Variety; *Processo*—Process; *Fermentação*—Fermentation; *Maceração carbônica*—Carbonic maceration; *Região*—Region; *Sul de Minas* (*Gerais*)—South of Minas (Gerais).

The labels were printed on matte adhesive paper and applied on the front side of the packages. The packages were coffee pouch bags from identical material (light pink polyethene [46]) and of identical size. The colour pink was chosen because it was demonstrated to be associated with desirable attributes of specialty coffee, both sensory (e.g., sweet, fruity, floral, light roast) and conceptual (i.e., modern). The bags were filled with 200 g of roasted coffee before being sealed. In addition to the information about the process, all three labels displayed the same written information using the same typeface and also featured the same design (Figure 1).

### 2.3. Design and Procedure

The experiment was conducted at Sofa Cafe, a coffee shop and school in Sao Paulo, Brazil. The use of contexts that are realistic enough and relevant to consumers is an important aspect of consumer research. Ecologically valid paradigms (i.e., data acquisition under more naturalistic testing conditions) generate results more readily applicable to real-world situations—compared to traditional laboratory methods [47,48]. The sessions were carried out over a weekend (from 9 am to 5 pm) and participants were recruited through social media or as customers/clients of the shop. The online advert made clear the experimental procedure as well as the inclusion criteria (only amateur consumers who had been drinking specialty coffee for at least a year with no sugar added, and who did not have a cold or any other impairment of their sense of smell or taste at the time of the study could take part). Before the start of the study, the participants were given the informed consent to read and sign and were asked to fill in a short questionnaire about demographics, their familiarity with and consumption frequency of specialty coffee.

The participants were led, in groups of eight, into a quiet, well-lit and air-conditioned testing room and were then seated at a table with at least 1 m spacing between adjacent tasters. At the start of the tasting session, an evaluation sheet containing the ‘expectation’ and ‘perception’ rating scales and a glass of water were placed in front of each participant. Then, the group of participants received a three-minute briefing in order to ensure that all groups were given the same instructions.

The participants were told they were going to evaluate three coffee samples paired with their respective packaging, one at a time. For each sample, the assessment started by placing a coffee bag in front of the participants. They were instructed to look at, but not touch or smell, the coffee bag and rate their expectations of aroma, flavour, acidity, and sweetness as well as how much they expect to like the coffee inside using the ‘expectation’ rating scales. Once the pre-tasting test was completed, the participants were served approximately 60 mL of brewed coffee in white porcelain cups. Next, they were asked to taste the coffee sample and evaluate their perception of aroma, flavour, acidity, and sweetness using the ‘perception’ scales. Finally, they judged how much they liked the coffee and their intention to purchase it. Ratings were performed using a 10 cm visual analogue scale anchored at 0 (“not at all”) and 10 (“very”). VAS was selected over a structured scale (with numeric and/or semantic labels) to avoid non-normal data distribution due to clustering near the labels (i.e., categorial behaviour) [49].

The tasting procedure followed a within-subject experimental design, and the evaluation of the samples followed a sequential monadic presentation scheme with the order of presentation balanced amongst participants. Thus, the same procedure was repeated three times, one for each coffee bag/label. The participants were also instructed to rinse their mouths out with water between samples in order to cleanse their palates. The tasting session lasted for around 20 min.

After they completed the tasting session, they were asked to respond to a short questionnaire regarding their impressions about post-harvest process information presented on specialty coffee labels. They started by answering a yes/no question about whether they consider the information about the coffee post-harvest process relevant for their coffee choice. If they answered yes, the next question presented a list of five different post-harvest processes, namely, ‘natural’, ‘washed’, ‘yeast fermentation’, ‘anaerobic fermentation’, and ‘carbonic maceration’. Natural and washed are traditional fermentation methods used in coffee post-harvest whereas carbonic maceration as well as fermentation using yeast as starter and anoxic environment are innovative techniques [3]. The selection of terms was based on how the information about post-harvest is usually presented to the end consumer on specialty coffee labels [50]. For each process, the participants were asked to rate how usual/ordinary or exotic they expect the coffee’s overall flavour to be using a continuous ‘usual-to-exotic’ bipolar scale anchored at the polar terms ‘totally usual’ (0) and ‘totally exotic’ (10). Finally, they were asked to select which coffee process they considered to have the highest price (most expensive) and, conversely, which coffee they considered to have the lowest price (cheapest).

### 2.4. Data Analysis

The data was analyzed using repeated measures, multivariate analysis of variance (RM-MANOVA) on the dependent variables ‘aroma’, ‘flavour’, ‘acidity’, ‘sweetness’, and ‘liking’ for expected coffee ratings (pre-tasting). The RM-MANOVA was also performed on the post-tasting data in order to assess the participants’ actual perception of aroma, flavour, acidity, sweetness, and on their ‘liking’ and ‘purchase intent’ ratings (post-tasting). The information type (no information, fermentation, carbonic maceration) was the independent variable.

Additionally, a repeated-measures MANOVA was used to compare the expected coffee ratings to the post-tasting ratings on the dependent variables ‘aroma’, ‘flavour’, ‘acidity’, ‘sweetness’, and ‘liking’, where rating type (pre- or post-tasting) was included as an additional within-participants factor. All post hoc pairwise comparisons were Bonferroni corrected, and differences were considered significant at *p* ≤ 0.05.

The data from the ‘usual-to-exotic’ bipolar scale was also analyzed using RM-MANOVA, having the processes as independent variables and the ‘usual-to-exotic’ continuum as the dependent variable. For the lowest and highest price data, frequency was calculated and the values were recoded as dichotomous. Cochran Q tests were performed to test for differences amongst the processes selected as being the cheapest and also differences amongst the processes selected as being the most expensive.

The statistical analyses were performed using the IBM SPSS version 22.0 (IBM Corporation, Armonk, NY, USA).

## 3. Results

### 3.1. Tasting Session

The RM-MANOVA test revealed a significant main effect of information type (no information, fermentation, carbonic maceration) on participants’ ratings in both pre-tasting [F(3,177) = 29.76, *p* < 0.01, Wilks’ lambda = 0.36; η^2^*_p_* = 0.64] as well as post-tasting [F(3,177) = 13.07, *p* < 0.01, Wilks’ lambda = 0.52; η^2^*_p_* = 0.48] assessments.

#### 3.1.1. Taste Expectation (Pre-Tasting Ratings)

Further univariate ANOVAs revealed significant effects of information type on the expectation of flavour [F(2,178) = 11.94, *p* < 0.01, η^2^*_p_* = 0.06], acidity [F(2,178) = 111.51, *p* < 0.01, η^2^*_p_* = 0.40], sweetness [F(2,178) = 20.65, *p* < 0.01, η^2^*_p_* = 0.10], and liking [F(2,178) = 33.61, *p* < 0.01, η^2^*_p_* = 0.16] ratings. No significant effect of information type on expectation of aroma intensity was observed (*p* = 0.08).

**Figure 2:**
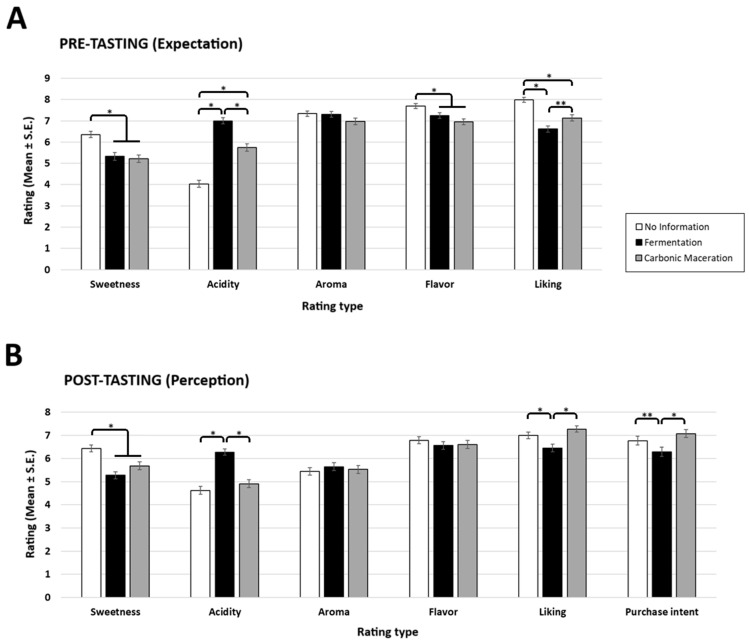
Mean ratings (±SE) of (**A**) pre-tasting (expectation) and (**B**) post-tasting (perception) as a function of rating type (sweetness, acidity, aroma, flavour, liking, purchase intent) for all three label information types (no information, fermentation, carbonic maceration). Asterisks indicate statistical significance at *p* < 0.01 (*) or *p* < 0.05 (**) (Bonferroni corrected).

Additional pairwise tests (Bonferroni corrected) showed specific effects for each information type. The coffee from the bag with no information (NI) about the fermentative post-harvest process was expected to be sweeter, more intense in flavour, more liked, and less acidic than the coffees from the bags labelled as fermentation (F) and carbonic maceration (CM) (all *p* < 0.05). Additionally, the coffee from the carbonic maceration bag was expected to be less acidic and also more liked than the coffee from the fermentation bag (all *p* < 0.05) (flavour: NI = 7.7 ± 0.1; F = 7.3 ± 0.1; CM = 6.9 ± 0.1; acidity: NI = 4.0 ± 0.2; F = 7.0 ± 0.1; CM = 5.8 ± 0.2; sweetness: NI = 6.3 ± 0.2; F = 5.3 ± 0.2; CM = 5.2 ± 0.2; liking: NI = 8.0 ± 0.1; F = 6.6 ± 0.2; CM = 7.1 ± 0.1) (Figure 2A).

#### 3.1.2. Taste Perception (Post-Tasting Ratings)

Univariate ANOVAs showed that information type exerted a significant effect on participants’ ratings of acidity [F(2,178) = 43.12, *p* < 0.01, η^2^*_p_* = 0.20], sweetness [F(2,178) = 18.66, *p* < 0.01, η^2^*_p_* = 0.09], liking [F(2,178) = 13.83, *p* < 0.01, η^2^*_p_* = 0.07], and purchase intent [F(2,178) = 6.94, *p* < 0.01, η^2^*_p_* = 0.04]. There was no significant effect of information type on the perception of aroma (*p* = 0.56) and flavour (*p* = 0.39).

Further post hoc comparison (Bonferroni corrected) revealed that the coffee paired with the bag with no information on fermentation process was perceived as being sweeter than the other two coffee samples (all *p* < 0.01) (sweetness: NI = 6.4 ± 0.2; F = 5.2 ± 0.2; CM = 5.7 ± 0.2). Moreover, the coffee paired with the bag labelled as ‘fermentation’ was rated as more acidic, was less liked, and was given lower ratings of purchase intent than the other coffees (all *p* < 0.01) (acidity: NI = 4.6 ± 0.2; F = 6.3 ± 0.1; CM = 4.9 ± 0.2; liking: NI = 7.0 ± 0.1; F = 6.4 ± 0.2; CM = 7.3 ± 0.1; purchase intent: NI = 6.8 ± 0.2; F = 6.2 ± 0.2; CM = 7.1 ± 0.2) (Figure 2B).

#### 3.1.3. Taste Expectations Versus Taste Perception

The repeated RM-MANOVA revealed significant main effects of rating type (pre- and post-tasting) [F(1,179) = 43.33, *p* < 0.01, Wilks’ lambda = 0.44; η^2^*_p_* = 0.55] and information type [F(2,178) = 32.27, *p* < 0.01, Wilks’ lambda = 0.34; η^2^*_p_* = 0.65] as well as a significant interaction between the two factors [F(3,177) = 9.29, *p* < 0.01, Wilks’ lambda = 0.65; η^2^*_p_* = 0.35] on participants’ ratings. Univariate ANOVA showed that, overall, the coffees were expected to be more aromatic (pre: 7.2 ± 0.1; post: 5.5 ± 0.1), more flavourful (pre: 7.3 ± 0.1; post: 6.6 ± 0.1), and more liked (pre: 7.3 ± 0.1; post: 6.9 ± 0.1) than they were actually perceived as being (all *p* < 0.01).

**Figure 3:**
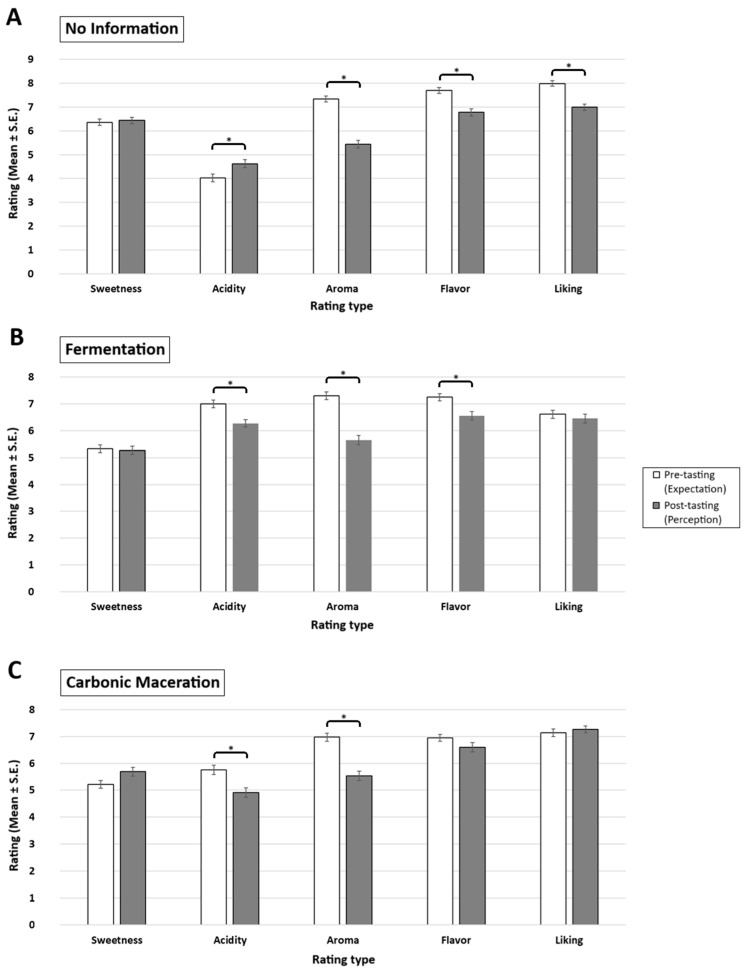
Mean ratings (±SE) of pre-tasting (white), post-tasting (grey) as a function of rating type (sweetness, acidity, aroma, flavour, liking) and the label information type: (**A**) No information, (**B**) Fermentation, (**C**) Carbonic maceration. *Asterisks* indicate statistically significant difference between pre- versus post-tasting ratings at *p* < 0.01 (*) (Bonferroni corrected).

As per the interaction effects, further ANOVAs and post hoc comparisons showed specific effects for each information type (Figure 3). For the no information condition, the coffees were expected to be less acidic (pre: 4.0 ± 0.2; post: 4.6 ± 0.2), more aromatic (pre: 7.3 ± 0.1; post: 5.5 ± 0.1), more flavorful (pre: 7.7 ± 0.1; post: 6.8 ± 0.1), and more liked (pre: 8.0 ± 0.1; post: 7.0 ± 0.1) than they were actually perceived as being (all *p* < 0.01). For the fermentation condition, the coffees were given higher ratings of expected acidity (pre: 7.0 ± 0.1; post: 6.3 ± 0.1), aroma (pre: 7.3 ± 0.1; post: 5.7 ± 0.1), and flavour (pre: 7.3 ± 0.1; post: 6.6 ± 0.1) compared to the actual perception ratings (all *p* < 0.01). Finaly, for the carbonic maceration condition, the coffees were expected to be more acidic (pre: 5.8 ± 0.2; post: 4.9 ± 0.2) and more aromatic (pre: 7.0 ± 0.1; post: 5.5 ± 0.1) than they were actually perceived as being (all *p* < 0.01) (Table 1; Figure 3).

### 3.2. Expected Overall Flavour Characteristics and Pricing

**Figure 4:**
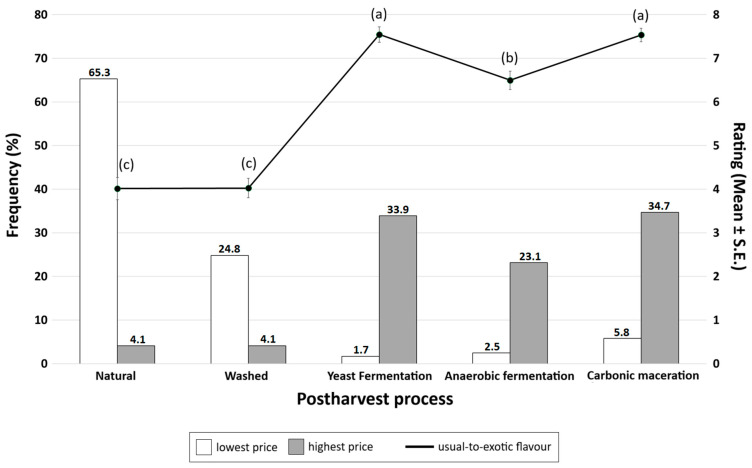
Relationship between attributed expensiveness (grey bars) and expected exotic flavour profile (black line) of coffees from different post-harvest processes. Percentage frequency distribution of the processes considered to have the lowest (white bars) and highest prices (grey bars) by participants are shown in the left Y axis. Rating of the expected overall flavour profile (black line) for each process using a continuous ‘usual-to-exotic’ bipolar scale is shown in the right Y axis. Values with different letters (a, b, c) are significantly different from one another (*p* < 0.01).

The RM-MANOVA test showed a significant effect of the post-harvest process on the expectation of the overall flavour characteristics of the coffee [F(2,178) = 18.66, *p* < 0.01, η^2^*_p_* = 0.09]. Further pairwise tests (Bonferroni corrected) showed that ‘yeast fermentation’ (7.5 ± 0.2) and ‘carbonic maceration’ (7.2 ± 0.2) coffees were expected to have the most exotic flavour, followed by ‘anaerobic fermentation’ (6.8 ± 0.2), and the least exotic—or the most usual—flavour is expected from coffees processed as natural (4.0 ± 0.3) and washed (4.0 ± 0.2) (all *p* < 0.01) (Figure 4).

A total of 121 (67.2%) consumers reported that the information about the coffee post-harvest process is relevant to their coffee choice. For these 121 consumers, the carbonic maceration process was considered the most expensive by 34.7% (*n* = 42) of consumers, followed by yeast fermentation (33.9%; *n* = 41), anaerobic fermentation (23.1%; *n* = 28), and natural and washed (equally 4.1%; *n* = 5). Conversely, 65.3% (*n* = 79) of participants considered the natural process to have the lowest price, followed by washed (24.8%; *n* = 30), carbonic maceration (5.8%; *n* = 7), yeast fermentation (2.5%; *n* = 3), and anaerobic fermentation (1.7%; *n* = 2) methods (Figure 2). Cochran’s Q tests determined that there was a statistically significant difference in the proportion of selected processes for both the highest [χ^2^(4) = 96.28, *p* < 0.01] and lowest [χ^2^(4) = 319.42, *p* < 0.01] prices.

## 4. Discussion

The present study was designed to investigate whether the label term used to describe the post-harvest fermentative process would influence participants’ expectation and/or perception of brewed coffee. During coffee tasting sessions, three packaging labels that provided different post-harvest information (‘no information’, ‘fermentation’, ‘carbonic maceration’) were paired with coffee samples and presented to specialty coffee consumers. The consumers first evaluated their expectations about the coffees inside the bags (pre-tasting), and then went on to taste and rate the coffees based on perceived sensory attributes, liking, and purchase intent (post-tasting). Additionally, consumers were given a list of five post-harvest processes that included traditional techniques (natural and washed processing) as well as innovative and precision methods (yeast fermentation, anaerobic fermentation, and carbonic maceration), all commonly found on specialty coffee labels. They were asked to rate their expectations regarding usual-to-exotic coffee flavour and also indicated which of those processes they considered to be the most expensive as well as the cheapest.

The bag label paired with the coffee sample affected the expectation and subsequent perception of acidity and sweetness, as well as the judgements of liking and purchase intent, by specialty coffee consumers.

In terms of acidity, as hypothesized, the coffee paired with the bag labelled ‘fermentation’ was expected to have the highest acidity and also to be the least liked compared to the other two conditions. This expectation was confirmed during tasting, as the ‘fermentation’ coffee sample was indeed perceived as the most acidic and was least liked by participants. It also received the lowest ratings for purchase intent. The coffee paired with the ‘carbonic maceration’ bag was also expected to have higher acidity and to be less liked than the control, but also to be less acidic and more liked than the ‘fermentation’ condition. Interestingly, the post-tasting ratings revealed that the ‘carbonic maceration’ coffee did not show any differences from the control in terms of perceived acidity, liking, and purchase intent. Regarding sweetness, the coffee from the bags labelled ‘fermentation’ and ‘carbonic maceration’ were anticipated to be less sweet than the coffee from the ‘no information’ bag. These expectations were transferred over to affect the actual perception of sweetness, as both coffee samples were rated significantly lower in sweetness compared to the control sample.

These findings are in agreement with previous studies showing that the sensory expectations—and subsequent perception and liking—of a product can be greatly influenced by extrinsic cues [20]. Under the broad umbrella of extrinsic cues, which can range from packaging and serving ware to the location where the food is sold or consumed, there are verbal terms used to describe the food. It has been known for many years that food names and descriptive terms can change what people say about the taste, flavour, and/or aroma of a food and how much they like it, not to mention how much they are willing to pay for it (e.g., [51,52,53,54]).

In the present study, consumers were served the exact same brewed coffee in all three conditions; the only difference was the label information presented alongside the coffee samples. The coffee believed to have undergone post-harvest ‘fermentation’ was perceived to have such an intense level of acidity that it significantly affected its acceptance negatively. In fact, in the case of the development of new fermented foods and beverages, the sensory properties represent one of the most important drivers of consumer perception and preference [33]. For instance, ref. [55] showed that for a watermelon juice fermented with lactic acid bacteria, the descriptors considered as ‘natural characteristics’ (e.g., sweet, watermelon flavour) were the most appreciated by consumers; on the contrary, descriptors of sour and bitter were the least appreciated. Similarly, it has been shown that fermented coffees tend to be perceived with significantly strong acidity and grassy/vegetable notes by consumers, which has been negatively correlated with acceptance and emotional response measures [56,57]. Additionally, the perception of heightened levels of acidity can diminish the perception of sweetness, an effect that has also been demonstrated in cold brewed coffee [58].

When a familiar product, like watermelon juice or specialty coffee, is expected to have mild acidity by a consumer, an acidity level that is perceived to be too high can create a dissonant flavour experience. In the aforementioned studies about fermented watermelon juice and coffee, flavour perception and acceptance were tested in blind tasting sessions. However, in our study, the information about ‘fermentation’ was disclosed to the participants before tasting, which influenced their expectation towards acidity. Thus, even though the perceived acidity level was congruent with the expectation triggered by the ‘fermentation’ label, it seems to have been incongruent with the consumers’ preconceived expectation of coffee as a familiar product. This indicates that the tasted level of acidity for the coffee sample, even being a ‘fermented’ one, fell outside of consumers’ general expectations for the magnitude of attributes that a coffee can contain [24,59].

According to the predictive processing framework, it can be argued that the consumers’ brains must make a call about whether the prediction of what a ‘fermented coffee’ should taste like and the incoming sensory signals can be matched. It will initially tend to follow the assimilation model by trying to minimize the error (or discrepancy) and adjust the sensory input to the internal prediction. But if the error signal is large enough, it will result in a noticeable disconfirmation of expectations and the contrast will occur [24,27]. Additionally, a large prediction error can cause the consumer to exaggerate the discrepancy between their expectations and reality, leading to a product evaluation that shifts in the opposite direction from what they originally anticipated [28].

Interestingly, the effect observed for the label ‘carbonic maceration’ differed from those of the label ‘fermentation’. The ‘carbonic maceration’ label led to expectations that the coffee would be less acidic and more liked than the ‘fermentation’ one, but would also be significantly more acidic and less liked than the control. During the post-tasting, the ‘carbonic maceration’ coffee did not show any differences from the control in terms of perceived acidity, liking, and purchase intent. Despite the fact that the term ‘carbonic maceration’ somewhat enhanced the expected acidity of the coffee compared to the control, this expectation did not carry over to influence the actual tasting experience. A striking demonstration of how the label of a food can play a crucial role in setting expectations towards a familiar looking food (i.e., a pink ice-cream [45]). Three different groups of participants were served a scoop of a pink ice-cream to taste under three different labels, namely, ‘ice-cream’, ‘savoury mousse’, and ‘Food 386’, respectively. Other than the label description, none of the groups were informed on the actual food they were about to taste: a smoked-salmon ice-cream. Labelling the food as ice-cream generated strong expectations of a sweet, fruity flavour, consistent with the visual appearance of the pink ice-cream, which resulted in higher ratings of saltiness and lower ratings of pleasantness of the food. Contrarily, the uninformative label ‘Food 386’ was associated with acceptable levels of perceived saltiness and higher ratings of pleasantness. Our results followed a similar pattern in the sense that the perception and acceptance of the same coffee sample was highly dependent on the label description presented when the coffee was served. The term ‘fermentation’ is accurate whilst the term ‘carbonic maceration’ is vague and uninformative, and this distinction led to different tasting experiences regarding the expected and perceived intensity of acidity as well as liking. This effect can be related to the curiosity hypothesis ([60]; see also [61]). According to this hypothesis, small deviations from the level that the consumer has adapted to can be seen as interesting and as a novelty, thereby sparking the consumer’s curiosity. They are less likely to evoke negative hedonic changes. The consumer’s curiosity can be triggered by novel and/or uncertain terms in labels, which will in turn reduce the expectation (and subsequent surprise), and the experience is likely to be perceived as pleasant [62].

It is also worth noting that the term ‘fermentation’ on product labels can affect consumer acceptance for reasons beyond expected and/or perceived acidity [33]. For instance, it has been shown that sociocultural factors can impact consumers’ attitudes towards fermented foods due to associations with undesirable flavours or concerns about food safety, especially in cases of traditional beverages [63]. Whilst these sociocultural influences were not directly explored in our study, they are important factors to consider when interpreting the results.

In addition to the tasting session, consumers were presented a list of traditional and innovative fermentation methods, and asked to rate the expected overall flavour of these coffees, from usual to exotic. They were also asked to select the coffee they perceived to be the most and the least expensive from the provided list. As expected, the innovative fermentation-related terms ‘carbonic maceration’, ‘yeast fermentation’, and ‘anaerobic fermentation’ were considered as being more expensive and having more exotic flavours than the traditional fermentation methods ‘natural’ and ‘washed’. Studies on beer [64], wine [65], and dairy [66] have shown that consumers associate innovative fermentation techniques with advanced technologies as well as exotic flavour. This can lead to a perception that the product requires more effort and incurs higher costs to be produced, justifying a higher price. This finding also supports prior observations that consumers tend to associate foods made using more complex and innovative processes with higher quality and unique flavours [34,35].

When we compare the results from the tasting session and the short questionnaire, an interesting observation emerges. The short questionnaire showed that the consumers did perceive coffees produced with fermentative techniques as being more expensive and having unique flavours. Nonetheless, during the tasting session, the coffee paired with the label ‘fermentation’ was the least liked and the one that received the lowest ratings of purchase intent. Indeed, it is known that several factors influence consumer perceptions, preferences and purchase decisions towards a food product, with the sensory properties the major driver of food choice (e.g., [67]).

It is essential to acknowledge the limitations of this study. We specifically targeted Brazilian specialty coffee consumers, as Brazil is both the world’s largest producer and the second-largest consumer of coffee, representing a key market for specialty coffee. Whilst our findings offer valuable insights, they should be applied cautiously to other Western cultures, and cross-cultural differences in labelling and preferences must be considered. Furthermore, although a single tasting session can provide preliminary data on how labels affect preference and choice, it may not accurately predict long-term consumption habits, which are shaped by more complex and repeated interactions with the product.

## 5. Conclusions

Our findings provide evidence that expectations play a major role in generating sensory and hedonic responses to food stimuli. We observed that the same coffee received higher or lower liking ratings depending on whether the term ‘fermentation’, as a description of post-harvest process, was present on the bag label. At the same time, innovative post-harvest methods, which can include the term ‘fermentation’, were perceived as more expensive and associated with more exotic flavours compared to traditional post-harvest methods. These results contribute to the extensive literature demonstrating that people’s response to food is to a significant extent driven by the way a food product is described, including labelling terms. As consumers become more focused on quality and more aware of the production processes and ethical implications of their food, there is a rising demand for coffee products with informative labelling. However, as a practical implication, it is important to consider that certain descriptive names of production processes can negatively influence consumers’ acceptance and purchase intentions. Appropriate language use, such as choosing names with positive rather than negative associations, can effectively promote new technologies in consumer communication.

## Figures and Tables

**Table 1:** Compilation of pre- and post-tasting aroma, flavour, sweetness, acidity, liking, and purchase intent ratings (mean ± SE) across all three information conditions.

Measure	No Information (Mean ± SE)		Fermentation (Mean ± SE)		Carbonic Maceration (Mean ± SE)	
	Pre-Tasting	Post-Tasting	Pre-Tasting	Post-Tasting	Pre-Tasting	Post-Tasting
**Aroma**	7.3 ± 0.1	5.5 ± 0.2	7.3 ± 0.1	5.7 ± 0.1	7.0 ± 0.1	5.5 ± 0.1
**Flavour**	7.7 ± 0.1	6.8 ± 0.1	7.3 ± 0.1	6.6 ± 0.1	6.9 ± 0.1	6.6 ± 0.2
**Acidity**	4.0 ± 0.2	4.6 ± 0.2	7.0 ± 0.1	6.3 ± 0.1	5.8 ± 0.2	4.9 ± 0.2
**Sweetness**	6.3 ± 0.2	6.4 ± 0.2	5.3 ± 0.2	5.3 ± 0.1	5.2 ± 0.2	5.7 ± 0.2
**Liking**	8.0 ± 0.1	7.0 ± 0.1	6.6 ± 0.2	6.4 ± 0.2	7.1 ± 0.1	7.3 ± 0.1
**Purchase intent**	-	6.8 ± 0.2	-	6.2 ± 0.2	-	7.1 ± 0.2

## Data Availability

The original contributions presented in this study are included in the article. Further inquiries can be directed to the corresponding author.
